# Mr. Browne's Case of Wounded Artery

**Published:** 1809-04

**Authors:** John Browne

**Affiliations:** Surgeon. Plymouth Dock


### *To the Editors of the Medical and Physical Journal.*

GENTLEMEN,

AMONG the many alarming cases, wherein immediate danger is apprehended, and to which the surgeon stands hourly exposed to be called on for the exertion of every talent and skill that surgical aid can afford, I think none more important than that of wounded arteries. A case of this nature occurred, to which I was called; and it having terminated contrary to the theory of some of our first anatomists, I am led therefrom, to communicate it to the Medical World.

Oct. 13, 1808. In the evening I was called to a Captain Broderick, aged 28 years, at an inn in this neighbourhood, whom, on entering the room, I saw on the floor, sitting in a bent position (supported), and no doubt, thereby, making a considerable pressure on the wound; he was quite exhausted from loss of blood, which, from appearance, had been very considerable. On inquiry, I was informed by many who had now assembled, that he had been stabbed by a Portuguese refugee; I thereupon immediately examined the body, and found a wound in the upper part of the left thigh, into which I instantly put my finger, while he was raised on a table, and had his pantaloons, boots, &c. cut off. On further examination, I found the wound situated near two inches below Poupart's ligament, in the course of the femoral artery; which, with the immense loss of blood that had already taken place, convinced me that that artery was wounded; the only step I had consequently to take for the preservation of my patient, was that of securing the vessel, which, with the kind assistance of my friends Messrs. Dunning and Tripe, surgeons of this town, (who fortunately were by this time arrived) I effected in the following manner. Having made a very effectual compress with a common door key, on the external iliac artery, immediately as it emerges from the abdomen, I dilated with a scalpel the wound already inflicted by making an incision through the integuments three inches downwards, in the direction of the femoral artery; and upwards, as high as Poupart's ligament; next dividing the fascia, where the artery passes over the pectinalis muscle, which brought it with its cellular membrane to view, from which I carefully separated it. I then passed underneath it, (taking care not to include the nerve) a silver-eyed probe bent, armed with a waxed ligature of six threads, about one inch above the wound, and another lesser one below; tying each firmly with a common double knot. A trifling hæmorhage still continued from some of the veins; the instrument whereby the original wound was given having perforated the thigh to some depth beyond the artery. But we did not consider this hæmorrhage of sufficient importance to delay dressing of the wound; I therefore cleared it of all coagulum, and brought the edges together with three sutures, supported with adhæsive straps, avoiding all bandage, as I feared it might impede the circulation through the anastomosing branches. The operation being over, we had our patient placed in a warm *bed*, the limb being supported by pillows in a relaxed position. The surface of the body was at this time extremely cold; the pulse was scarcely perceptible; and all hæmorrhage had ceased. Four hours afterwards, the pulse being very feeble and quick, I gave him an anodyne, and left him in care of my young pupil for the night, from whom, on visiting him in the morning with my above medical friends,* I found

* Both these gentlemen expressed every anxiety for the success of the case, and continued their visits daily with me, until after the second operation.

I found he had been very restless, with frequent vomiting. The pulse had now increased in strength, and the heat of the thigh six inches below the wound, 80 by Fahrenheit's scale, gradually increasing for the first fifty hours to the knee; all below remaining cold. I then prescribed ol. Ricini, and ordered moderate friction, and warm spirituous fomentations to the leg and foot. Repeated his anodyne, and directed a farinaceous diet.

15. Visited him in the morning; pulse 100; had slept four hours; skin moist, tongue coated; had two evacuations; complained for the first time of slight pain in the wound, and extreme coldness; and partial loss of feeling in his leg and foot. Observed a circumscribed discoloured spot, about the sise of a shilling, on the inside of the thigh, and about three inches above the knee. Continued the fomentations as before, with an anodyne in the evening.

16. Passed a tolerable night; pulse 100. Repeated the ol. Ricini; removed the dressings partly from the wound, which looked well; temperature of the thigh equal to the other, all below continuing cold; slight vesications appearing on the inner gastrocnemius, continued the fomentations and the anodyne for the evening.

17. Treatment, &c. the same.

18. Rested well, pulse 100; vesications increased; dressed the wound, discharge copious, but good. Dr. Remmett's assistance was called in; a yeast cataplasm was applied to the whole leg and foot.

19. Pulse 100; wound looked healthy; cut away the sutures; vesications were much increased, so that the leg and foot were become sphacelated. The cinchona was given, and increased as far as the stomach could bear it.

20. Treatment, &c. continued.

21. A line of separation began to take place immediately below the knee; pulse 100. Treatment continued.

22. Pulse 100; sweats considerable; the vitriolic acid was added to the cinchona; on dressing the wound the lower ligature came away.

23. Treatment, &c. continued with but little variation to the 1st of November, on which day the line of separation appeared to be completely formed; the circumscribed spot on the thigh was also gradually separating, and the sweats were considerably increased. On a full consultation, a further delay of amputation was, notwithstanding, deemed adviseable from the following considerations; the healing of the part where the wound was first inflicted, which was going on in a kindly manner; the dilatation of the anastomosing branches could not but be advancing continually, and every day the danger of hæmorrhage was lessening; nor was the general state of the patient's health such as to forbid delay; a diet somewhat more generous, however, was recommended.

Nov. 2 and 3. Regimen, &c. continued.

4. The symptoms of hectic fever were evidently increasing, the bowels were become loose, and the patient appeared to be losing ground; and it was now resolved that the amputation should be performed on the following day.

5. I amputated the thigh immediately above the sphacelated spot; the loss of blood during the operation was considerable from the small branches, which were so numerous as to require thirteen ligatures; on examining the removed limb, we found the sphacelated spot to be superficial. A small portion of the periosteum was denuded where the amputation took place.

6. Rested well, pulse about 100; on dressing the upper wound, (which looked well) the upper ligature came away.

7. The pulse from 90 to 100; little or no discharge from the upper wound. Continued the cinchona.

8. Pulse as yesterday; removed the dressing in part from the stump, and found it look healthy.

9. Treatment, &c. continued.

10. Pulse 90; removed all the dressings from the stump, which looked well, and partial adhesion had taken place; the original wound remained stationary for the first fourteen days after the amputation, the stump was however, healing, and his health was improving very fast.

December 18. The original wound is now healed; the stump is doing very well, except that we are waiting for a trifling exfoliation which must take place; the patient's health is completely re-established.

Having thus given a plain detail of this important case, I beg leave to offer a remark or two, to which I am more particularly induced, by the belief that there is no instance on record, where the femoral artery has been tied above the profunda in consequence of recent wound. From our late improvements in anatomical science, I was emboldened to operate with some confidence, for the anastomosing branches are found to be particularly large and numerous in those parts, and our knowledge of the efforts of nature, to supply the defects in cases of aneurism, was very encouraging; but the termination of this case has proved our hopes to be fallacious, and evidently demonstrates the insuffiinsufficiency of the inosculations to support the limb, where its great artery is obliterated above the profunda from recent wound; they being in a natural and undilated condition.

I am, &c.

JOHN BROWNE, Surgeon.

*Plymouth Dock.*

